# Multimodality imaging in recognizing and differentiating cardiac masses in a patient with cancer presenting with non-ST-elevation myocardial infarction

**DOI:** 10.1093/ehjimp/qyae110

**Published:** 2024-10-24

**Authors:** Vasileios Bouratzis, Lampros Lakkas, Christos Floros, Anna Lea Amylidi, Nikoleta Douskou, Ilektra Stamou, Katerina K Naka

**Affiliations:** Second Department of Cardiology, University of Ioannina Medical School, University Campus, Stavros Niarchos Avenue, Ioannina 45 500, Greece; Physiology Department, Faculty of Medicine, School of Health Sciences, University of Ioannina, Ioannina 45 110, Greece; Second Department of Cardiology, University of Ioannina Medical School, University Campus, Stavros Niarchos Avenue, Ioannina 45 500, Greece; Department of Medical Oncology, University Hospital of Ioannina, Ioannina 45500, Greece; Second Department of Cardiology, University of Ioannina Medical School, University Campus, Stavros Niarchos Avenue, Ioannina 45 500, Greece; Second Department of Cardiology, University of Ioannina Medical School, University Campus, Stavros Niarchos Avenue, Ioannina 45 500, Greece; Second Department of Cardiology, University of Ioannina Medical School, University Campus, Stavros Niarchos Avenue, Ioannina 45 500, Greece

**Keywords:** fistula, coronary artery fistula, multiple fistulas, NSTEMI, cardiac masses

**Figure:**
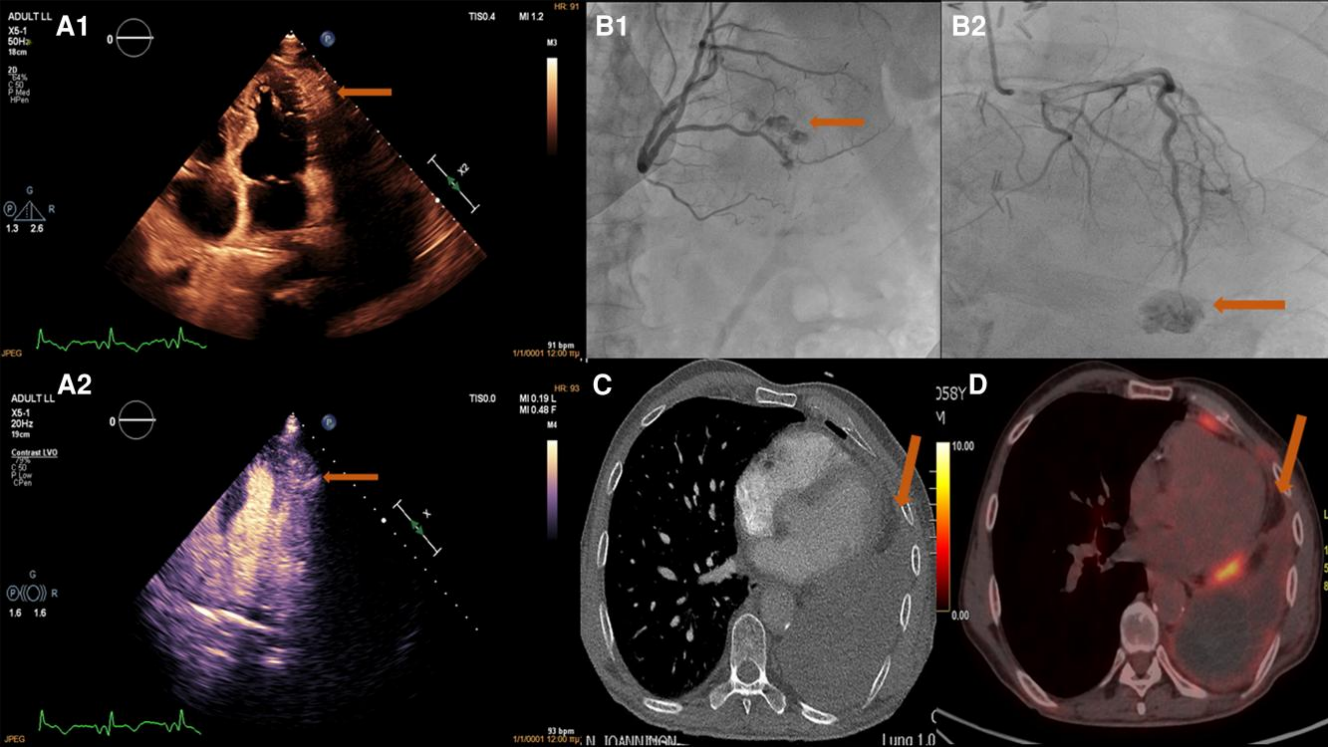


## Case description

A 58-year-old male presented with chest pain. He had a recent history of colectomy for rectosigmoid cancer. He also underwent a left pneumonectomy for two lung nodules: one was metastasis from the colon cancer, and the other was a primary squamous cell carcinoma of the lung. Electrocardiogram showed no dynamic changes, while troponin was elevated. A transthoracic contrast echocardiogram (due to the poor acoustic window) was performed that showed mildly reduced left ventricular (LV) systolic function and no significant valvular disease or pericardial fluid. A hyperechoic mass of circular/crescent shape and of inhomogeneous substance was revealed at the apical anterolateral LV wall that was enhanced using contrast medium (*Panels A1* and *A2*, Supplementary data online, *Video S1*). Our tentative diagnoses encompassed congenital or iatrogenic arteriovenous fistulas (including coronary fistulas) and cardiac vascular tumours (either benign such as haemangiomas or malignant such as metastatic lesions or angiosarcomas). Coronary angiography showed only a malformation with characteristic vascular blush in proximity to the distal left anterior descending artery, coming to confirm echocardiographic findings (*Panels B1* and *B2*, Supplementary data online, *Video S2*). A computed tomography (CT) pulmonary angiography was also performed, which excluded pulmonary embolism (there was no contrast enhancement during the venous phase of this CT—*Panel C*). A PET–CT scan was performed that failed to show hypermetabolism, thus excluding possible metastasis (*Panel D*). Since malignancy was excluded and our patient was asymptomatic with many comorbidities, a conservative treatment was decided.

Benign vascular cardiac malformations are either haemangiomas or coronary fistulas in most of the cases. Symptoms related to coronary shunting may be an indication for treatment, and in patients with cancer, a metastasis must be excluded (by using multimodality imaging) to make the final decision.

## Supplementary Material

qyae110_Supplementary_Data

## Data Availability

The data underlying this article are available in the article and in its online Supplementary material.

## Supplementary data

**Consent:** The authors confirm that written consent for submission and publication of this case report including images and associated text has been obtained from the patient in line with Committee on Publication Ethics guidance.

**Funding:** None declared.

## Lead author biography

Vasileios Bouratzis graduated from the University of Ioannina Medical School in 2016, and now he is a cardiologist trainee at the University Hospital of Ioannina, Greece. His main interests are coronary artery disease, cardiogenic shock, and cardioncology.

